# P-1313. A 10-year Trend on Antimicrobial Resistance of Pseudomonas aeruginosa from United States Medical Centers: Results from the INFORM Surveillance Program (2015-2024)

**DOI:** 10.1093/ofid/ofaf695.1501

**Published:** 2026-01-11

**Authors:** Helio SaderRodrigo E Mendes, John L Lock, Marisa Winkler, Mariana Castanheira

**Affiliations:** Element Iowa City (JMI Laboratories), North Liberty, IA; AbbVie Inc, Mettawa, Illinois; Element Materials Technology/Jones Microbiology Institute, North Liberty, Iowa; Element, North Liberty, IA

## Abstract

**Background:**

*P. aeruginosa* (PSA) represents an important cause of hospital-associated infection with high rates of antimicrobial resistance and elevated morbidity and mortality. We evaluated the antimicrobial susceptibility of PSA isolates from United States (US) medical centers in the last 10 years.

**Figure:**
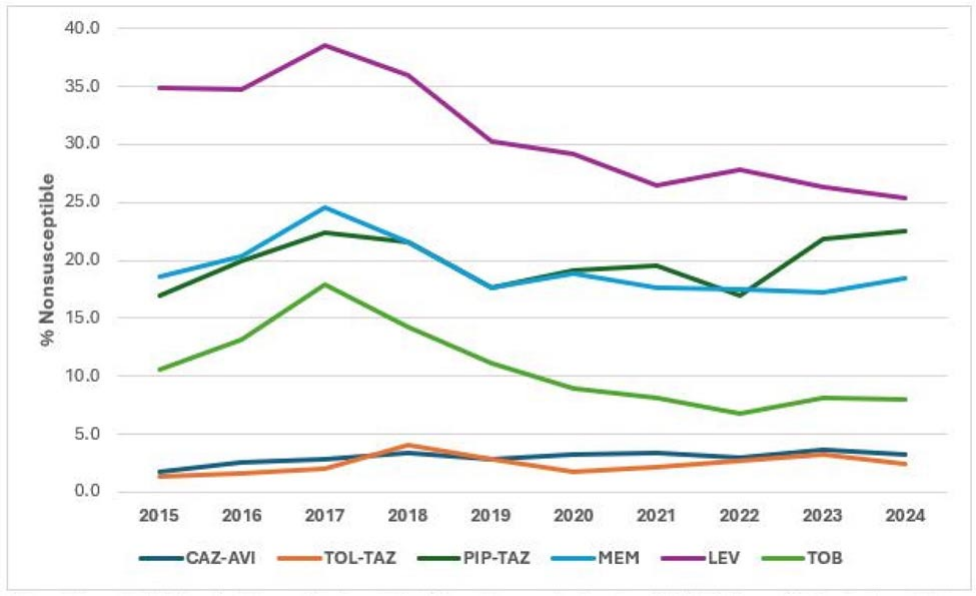
Frequency of Pseudomonas aeruginosa isolates nonsusceptible to selected antimicrobial agents Abbreviations: CAZ-AVI, ceftazidime-avibactam; TOL-TAZ, ceftolozane-tazobactam; PIP-TAZ, piperacillin-tazobactam; MEM, meropenem; LEV, levofloxacin; TOB, tobramycin.

**Figure:**
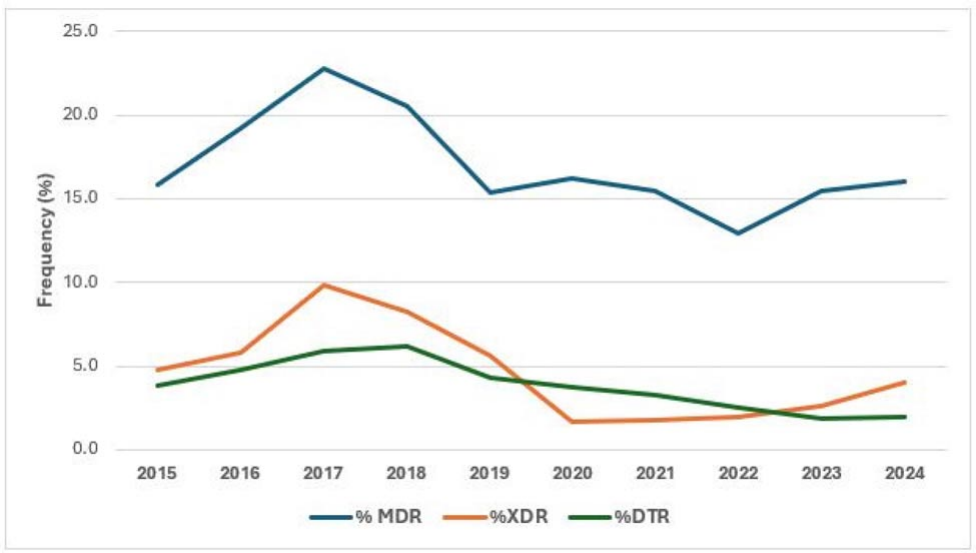
Frequency of isolates with multidrug-resistant (MDR), extensive drug-resistant (XDR), and difficult-to-treat (DTR) phenotypes

**Methods:**

14,305 PSA isolates (1/patient) were consecutively collected from 56 US medical centers in 2015-2024 and susceptibility tested against 17 antimicrobials by reference broth microdilution. Only centers that contributed to isolates for ≥ 8 years were included. Susceptibility results were stratified by year and infection type. Multidrug-resistant (MDR) was defined as resistance to ≥ 3 classes, extensively drug-resistant (XDR) as susceptibility to ≤ 2 classes and difficult-to-treat (DTR) as nonsusceptible (NS) to piperacillin-tazobactam (PIP-TAZ), cephalosporins, carbapenems, fluoroquinolones and aminoglycosides.

**Figure:**
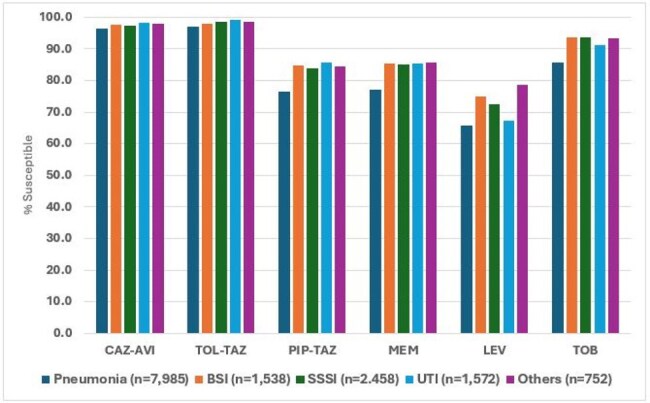
Susceptibility to selected antimicrobial agents stratified by infection type Abbreviations: CAZ-AVI, ceftazidime-avibactam; TOL-TAZ, ceftolozane-tazobactam; PIP-TAZ, piperacillin-tazobactam; MEM, meropenem; LEV, levofloxacin; TOB, tobramycin; BSI, bloodstream infection; SSSI, skin and skin structure infection; UTI, urinary tract infection.

**Results:**

The most active compounds were ceftazidime-avibactam (CAZ-AVI) and ceftolozane-tazobactam (TOL-TAZ) with overall susceptibility rates of 97.0% and 97.6%, respectively. NS rates for CAZ-AVI and TOL-TAZ increased from 1.8% and 1.4% in 2015 to 3.2% and 2.5% in 2024, respectively (Figure 1). PIP-TAZ NS rates increased from 17.0% in 2015 to 22.5% in 2024 with marked variation during this period. NS rates for meropenem (MEM), levofloxacin (LEV), and tobramycin (TOB) increased from 2015 to 2017 and then decreased progressively until 2023 or 2024. The frequency of MDR, XDR and DTR isolates increased until 2017, decreased from 2017 to 2022 or 2023, and then increased again in 2024 (Figure 2). CAZ-AVI was active against 83.0% of MDR, 66.0% of XDR, and 65.3% of DTR isolates, while TOL-TAZ was active against 85.9% of MDR, 64.3% of XDR, and 71.9% of DTR isolates. Susceptibility rates were lower among isolates from pneumonia compared to other infections (Figure 3).

**Conclusion:**

CAZ-AVI and TOL-TAZ remain very active against PSA from US hospitals, but susceptibility to these agents decreased slightly during the study period. PSA susceptibility to MEM, LEV, and TOB improved since 2017, with an accompanying reduction of the frequency of MDR, XDR, and DTR isolates until 2023-2024.

**Disclosures:**

Helio Sader, United States Food and Drug Administration: FDA Contract Number: 75F40123C00140 Rodrigo E. Mendes, PhD, GSK: Grant/Research Support|Shionogi & Co., Ltd.: Grant/Research Support|United States Food and Drug Administration: FDA Contract Number: 75F40123C00140 John L. Lock, PharmD, AbbVie: Stocks/Bonds (Public Company) Marisa Winkler, MD, PhD, Basilea: Advisor/Consultant|Basilea: Grant/Research Support|GSK: Advisor/Consultant|GSK: Grant/Research Support|Melinta Therapeutics: Advisor/Consultant|Melinta Therapeutics: Grant/Research Support|Mundipharma: Advisor/Consultant|Mundipharma: Grant/Research Support|Pfizer: Advisor/Consultant|Pfizer: Grant/Research Support|Pulmocide: Advisor/Consultant|Pulmocide: Grant/Research Support Mariana Castanheira, PhD, Melinta Therapeutics: Advisor/Consultant|Melinta Therapeutics: Grant/Research Support

